# P2Y6 Receptor-Mediated Spinal Microglial Activation in Neuropathic Pain

**DOI:** 10.1155/2019/2612534

**Published:** 2019-06-03

**Authors:** Jiang Bian, Ying Zhang, Yan Liu, Qun Li, Hai-bin Tang, Qing Liu

**Affiliations:** Department of Anesthesiology, Affiliated Traditional Chinese Medicine Hospital, Southwest Medical University, Luzhou 646000, Sichuan, China

## Abstract

**Objective:**

To explore the role of purine family member P2Y6 receptors in regulating neuropathic pain (NP) via neuroinflammation in the spinal cord.

**Methods:**

Chronic constriction injury of the sciatic nerve (CCI) of NP was classic in setting up models on Sprague-Dawley (SD) rats. Experiments were performed on rats with sham surgery, CCI, CCI + MRS2578 (a P2Y6 receptor antagonist), and UDP (a P2Y6 receptor agonist). The hyperalgesia intensity was mirrored by paw withdrawal threshold (PWT) and thermal withdrawal latency (TWL). Immunofluorescence staining and western blot were used to evaluate activated microglial marker Iba-1. Enzyme-linked immunosorbent assay (ELISA) was used to access levels of IL-6. Conventional reverse transcription polymerase chain reaction (RT-PCR) and western blot analysis were used to detect the expression of P2Y6 mRNA and activation of JAK/STAT signaling.

**Results:**

Among all groups, CCI caused decreased PWT and TWL compared to sham surgery, meaning a successful establishment of the NP model. These decreased values of PWT and TWL tests could be prevented by intraperitoneally injected MRS2578 and enhanced by UDP administration. Similarly, CCI induced increase of Iba-1 protein, P2Y6 mRNA expression, and circulating IL-6 secretion, as well as increased JAK2/STAT3 mRNA expression and phosphorylating modification in spinal cord tissues could also be diminished by MRS2578 treatment and exacerbated by UDP.

**Conclusions:**

These findings indicated the crucial role of the P2Y6 receptor in modulating the microglial and inflammatory responses in the process of NP *in vivo*. Results from this study would provide insights into targeting the P2Y6 receptor to treat NP in the near future.

## 1. Introduction

Neuropathic pain (NP) refers to pain evoked by disorders or injury of the nervous system, of which the specific mechanism remains unknown. Accordingly, the persistent and typical symptoms of NP (spontaneous pain, hyperalgesia, and allodynia) are refractory to analgesics, persecuting millions of people worldwide [1–3]. Therefore, it is urgent to explore the pathogenesis and therapeutic strategies for NP.

It is well established that neuroinflammation plays a vital role in the maintenance of NP, especially by mediating central sensitization [4, 5]. Importantly, the crosstalk between microglia and neurons represents a fundamental element underlying neuroinflammation, and inhibiting microglial activation could lead to pain alleviation, verified by previous researches [4, 6–9].

ATP receptors are widely scattered throughout the nervous system and participate in the glial neuroinflammatory response in facilitating microglial activation [9–12]. Among the ATP receptors, purinergic P2 receptors are divided into the ionotropic P2X receptor and G protein-coupled metabotropic P2Y receptors [13]. Previous studies have shown that P2X receptors are critical in NP due to the fact that they participate in the amplification of the nociceptive signal both in the peripheral nervous system and central nervous system [14]. However, there is little evidence that P2Y receptors play a role in NP in comparison to P2X receptors. The P2Y6 receptor is mainly present in spinal microglia, which is involved in the process of microglial activation [15]. Syhr et al. [16] proved that the P2Y6 receptor was upregulated in response to peripheral nerve injury, while another study also demonstrated that intrathecal administration of the P2Y6 receptor antagonist MRS2578 resulted in an antinociceptive role in the NP rat model, and the expression of microglial marker Iba-1 remarkably deceased in comparison to the untreated group [17].

In addition, other studies have shown that administration of the P2Y6 receptor antagonist could reduce the production of IL-6 [18, 19], which is a proinflammatory cytokine closely associated with the pathogenesis of inflammatory diseases [20]. Previous study indicated that the Janus tyrosine kinase (JAK) and signal transducer and activator of transcription (STAT) signaling pathway were situated in the downstream of the IL-6 receptor-mediated signaling pathway, activation of which triggered a series of events including microglia activation [21, 22]. Activated by stimulators, JAK proteins subsequently phosphorylate the STATs. STAT3, a member of the STAT family, has been shown indispensable for nociceptive transmission and microglial activation [22–26]. This JAK/STAT3 pathway is not only responsible for cellular communication for nociceptive modulator-like IL-6 but also known for modulating antinociceptive signaling downstream of IL-10 [21, 27]. Specific inhibition of the microglial JAK/STAT3 pathway could attenuate pain hypersensitivity and morphologic change of neurons in the spinal cord [28].

In this study, we aim to illustrate the role of the P2Y6 receptor in modulating the activation of the JAK/STAT pathway via upregulating IL-6. We have built a model of NP using Sprague-Dawley (SD) rats undergoing chronic constriction injury of the sciatic nerve (CCI) to analyze the dynamic expression of the P2Y6 receptor, IL-6, and JAK2/STAT3 pathway proteins during the development of NP. We have also determined the expression of microglial marker Iba-1 and corresponding morphological changes of microglial cells. Application of the P2Y6 receptor antagonist resulted in inhibition of microglial polarization and IL-6 production, leading to reduction of NP in CCI rats. Taken together, our research has shown the critical role of the P2Y6 receptor in modulating the JAK2/STAT3-mediated nociceptive transmission and microglial activation, which could act as a potential target for NP therapy in future clinical studies.

## 2. Materials and Methods

### 2.1. Animals

All experiments were performed on male Sprague-Dawley rats, weighing 220–250 g. All rats were obtained from Southwest Medical University Animal Laboratory Center (Luzhou, China) and bred in a 12 h light-dark cycle room with controlled temperature (25–27°C). All rats were given free access to food and water. All experiments were strictly carried out under the obligations of Institutional Animal Care and Use Committee of Southwest Medical University.

### 2.2. CCI Model and Groups

The methods of chronic constriction injury (CCI) model construction were conducted as previously described by Bennett and Xie [29]. In brief, rats were deeply anesthetized under the aseptic condition though intraperitoneal injection of 10% chloral hydrate (300 mg/kg). The skin was sterilized at the right midthigh level and freed from adhering tissue to expose the sciatic trifurcation. Four ligatures were loosely tied around the nerve trunk with about 1 mm spacing intervals with 4.0 silk. Then, the wound was closed layer by layer and disinfected with povidone iodine solution. In sham animals, the right sciatic nerve was exposed and without ligation. In the UDP group, the rats subjected to CCI were intraperitoneally injected with 1 ml UDP (100 nmol/ml) once a day until day 14 after surgery. As to the MRS2578 group, the CCI rats were intraperitoneal injected of 1 ml MRS2578 (1 *μ*mol/ml) once a day until day 14 after surgery.

### 2.3. Behavior Test

The paw withdrawal threshold (PWT) and thermal withdrawal latency (TWL) were tested at 1 day before (pre-1 d) and 3, 7, and 14 days after CCI surgery (post-3, -7, and -14 d). Von Frey filaments (Shanghai Yuyan Instruments Co., Ltd., Shanghai, China) were used to measure PWT. The rats were placed in a mesh cage plane for 5 min at first. Then, the ipsilateral midplantar surface of the right hind paw was perpendicularly pressed with a series of 7 von Frey filaments (2, 4, 6, 8, 10, 15, and 26 g, respectively). The paw bent slightly or left in place within 5 s was recorded as positive response. Every rat received ten tests by single filament before being pricked by the next larger filament. Each test was conducted with 5 min rest between intervals. One of the filaments caused at least six positive responses in ten stimulations was considered as the PWT.

Hot plate (Shanghai Yuyan Instruments Co., Ltd., Shanghai, China) was set to 52 ± 0.2°C for the TWL test. The thermal intensity remained unchanged throughout the experiment. The reaction time of thermal stimulation of the hind paw was recorded. Every rat received three tests, with an interval of 5 min between tests. The average of test results was used for TWL.

### 2.4. Sample Preparation

At the time point of pre-1 d and post-3, -7, and -14 d of CCI, five rats of each group were anesthetized with an intraperitoneal injection of 10% chloral hydrate (300 mg/kg) and quickly sacrificed by decapitation. Next, the L4-L5 spinal cord segments were dissected on ice before being transferred into the Eppendorf (EP) tubes preloaded with protease inhibitor (Baiyingbio, Shenzhen, China) and phosphorylase inhibitor (Baiaolaibo, Beijing, China) mixtures.

### 2.5. Immunofluorescence Assay

For immunofluorescence analysis, the L4-5 sections were fixed for 10 min with the acetone and washed with 0.01 M phosphate buffer saline (PBS) for 3 times (5 min each). After blocking with 10% normal goat serum in 0.01 M PBS + 0.3% Triton X-100 for 1 h at room temperature, the sections were incubated overnight at 4°C with the following primary antibody: rabbit anti-Iba-1 polyclonal antibody (1 :  500, Abcam, Cambridge, MA, United Kingdom). After rinsing with 0.01 M PBS for 3 times (10 min each), the sections were incubated with FITC-conjugated or Cy3-conjugated secondary antibodies (1 :  400, Jackson ImmunoResearch Laboratories Inc.) dissolved in 0.1% normal goat serum for 1 h at room temperature in dark. The sections were rinsed again and were subsequently stained with diamidino-2-phenylindole dihydrochloride (DAPI) (1 : 1,000; Beyotime Corp., Shanghai, China). Finally, the sections were rinsed with PBS, and the coverslips were mounted with glycerol solution. Negative staining controls were prepared by omitting either the primary antibody or secondary antibody. Fluorescent images were captured with a confocal microscope (model SP5 TCS; Leica, Heidelberg, Germany), and the fluorescence density was analyzed using Image-Pro Plus 6 (Media Cybernetics, USA).

### 2.6. Western Blot

Fresh tissues were subjected to homogenization, and total protein was extracted by the RIPA lysis buffer (Beyotime, Shanghai, China) mixed with proteinases. Protein concentration of the lysates was measured by the bicinchoninic acid (BCA) method (Beyotime, Shanghai, China). Samples were loaded onto the prepared 10% SDS-polyacrylamide gels (Beyotime, Shanghai, China) and separated by electrophoresis at 80 V for 30 min and then 120 V for 1 h. The separated proteins were transferred to a polyvinylidene difluoride (PVDF) membrane. Then, the membranes were blocked by a solution containing Tris-buffered saline with 0.05% Tween-20 (TBS-T) as well as 5% nonfat milk for 1 h, sequentially, probed (4°C; overnight) with one of the primary antibodies: rabbit anti-Iba1 IgG (1 : 500, Abcam, Cambridge, MA, United Kingdom), rabbit anti-p-JAK2 IgG (1 : 1000, Abcam, Cambridge, MA, United Kingdom), rabbit anti-p-STAT3 IgG (1 : 1000, Abcam, Cambridge, MA, United Kingdom), and rabbit anti-*β*-anctin for loading control (1 :  2000, Abcam, Cambridge, MA, United Kingdom). On the next day, the membranes were incubated with horseradish peroxidase (HRP) anti-rabbit secondary antibody (1 : 2000, ZSGB-BIO, ORIGENE, Beijing, China). Images were developed by the ECL plus reagent (P1010, Biotenology, Shanghai, China), and Gel-Pol analyzer (National Institutes of Health) was used to analyze the intensity of bands.

### 2.7. Reverse Transcription Polymerase Chain Reaction (RT-PCR)

L4-5 spinal cord tissues were taken out from the lumbar spine, and cords of each groups were put in 1 ml of the TRIzol Reagent (Invitrogen) and then stored at −80°C. According to the manufacturer's protocol, 40–70 mg of frozen tissue per sample was homogenized in the TRIzol Reagent (Invitrogen) by a hand-held homogenizer (Kontes Thomas Scientific, NJ). The total RNA concentration was measured by a spectrophotometer. The primer sequences of target genes and internal reference gene *β*-actin were designed using Primer Premier 5.0 software and then synthesized by Sangon Biotech (Shanghai). The primer sequences of this study were listed in Table 1. Total RNA was reverse transcribed into complementary DNA (cDNA) according to the instruction of QIAamp® MinElute® Virus Spin kit (Qiagen Inc USA). The reverse transcription was performed with the following program: 55°C for 10 s, 75°C for 20 s, and total of 40 cycles. Then, the cDNA PCR reaction system was transferred into the 7300 thermocycler (Applied Biosystems) with the following program: 95°C for 5 min, 95°C for 10 s, 55°C for 10 s, and 75°C for 20 s, and a total of 40 cycles. All values were normalized to *β*-actin expression for the loading control and then expressed as fold change with respect to mean controls values in the same run.

### 2.8. Enzyme-Linked Immunosorbent Assay (ELISA) for IL-6

1 ml of caudal vein blood samples were centrifuged (3000 r/min, 15 min). Serum was collected and quickly stored at −80°C. The concentrations of IL-6 in serum were measured spectrophotometrically by an ELISA kit following the manufacturer's instructions (BioSite, Paris, France).

### 2.9. Drugs

The P2Y6 receptor antagonist MRS2578 and agonist UDP were purchased from Tocris Bioscience (Ellisville, MO). The purinergic drugs referred above were selected according to relevant receptor selectivity and efficacy [30, 31]. MRS2578 and UDP were dissolved in 20% DMSO.

### 2.10. Statistical Analysis

SPSS 22.0 software (SPSS Inc., Chicago, IL, USA) was used for the statistical analysis. The quantitative data were presented as Mean ± SEM. Iba1 expression among four groups were analyzed with the one-way analysis of variance (ANOVA) followed by the Student–Newman–Keuls post hoc test. Other data were analyzed using two-way analysis of variance (ANOVA) followed by Holm–Sidak post hoc analysis. A value of *p* < 0.05 was considered statistically significant.

## 3. Results

### 3.1. Manipulation of P2Y6 Receptor-Modulated Sense of Pain in Neuropathic Pain (NP) Rat Models Induced by CCI

**Figure 1:**
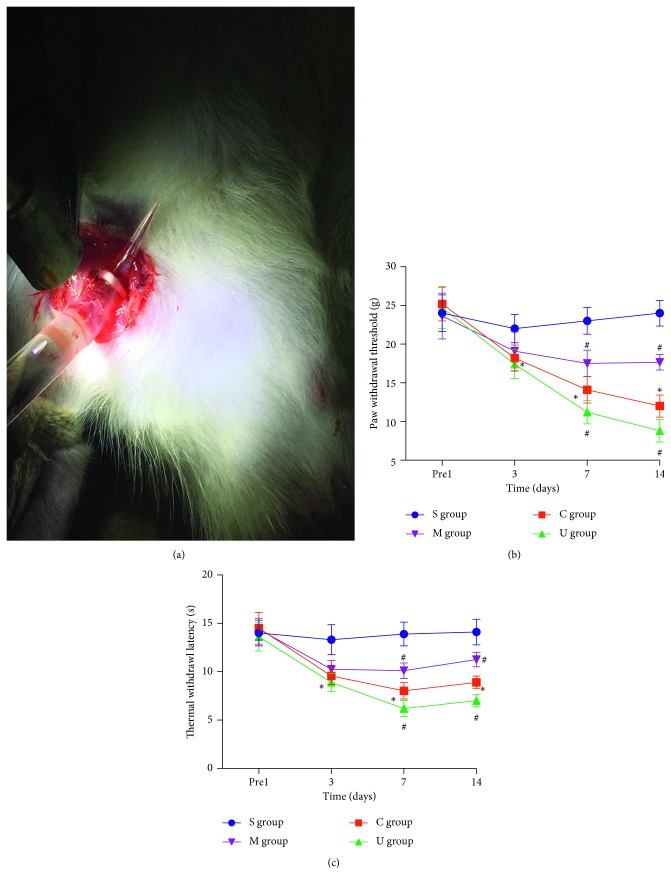
Manipulation of the P2Y6 receptor-modulated sense of pain in neuropathic pain (NP) in rat models induced by CCI. (a) The operation of CCI surgery. Changes of PWT (b) and TWL (c). Data (mean ± SEM) were presented in all rats. S: sham-operated rats; M: CCI rats treated with MRS2578; C: CCI rats; U: CCI rats treated with UDP. Significance of pain behavioral changes was analyzed with two-way ANOVA followed by Holm–Sidak post hoc analysis (^*∗*^*P* < 0.05, vs the sham group; ^#^*P* < 0.05, vs the CCI group).

To evaluate how P2Y6 might play a role in regulating NP in vivo, we established a NP animal model by chronic constriction injury of the sciatic nerve (CCI), as shown in Figure 1(a). By evaluating the data of paw withdrawal threshold (PWT) (Figure 1(b)) and thermal withdrawal latency (TWL) (Figure 1(c)), we found the CCI rats (red) gradually exhibited the typical symptoms of hyperalgesia and allodynia at 3, 7, and 14 d after CCI, while sham-operated rats (blue) showed no obvious changes at all time points. The PWT and TWL values of CCI rats remarkably decreased on day 3 after CCI and sustained to day 14 (*P* < 0.05), which meant NP had developed on day 3 and reached its peak on day 14. To test whether the P2Y6 receptor was a modulator in NP, we treated the CCI rats with a P2Y6 antagonist MRS2578 (purple) and a P2Y6 agonist UDP (green), respectively. After persistent intraperitoneal administration of MRS2578 at day 1 to day 14 after CCI, the hyperalgesia started to alleviate on day 7 till day 14 compared to that without treatment (red, *P* < 0.05). In contrast, treatment of UDP on CCI rats showed a greater value of PWT and TWL tests, an indicator of increased pain intensity, on day 7 and day 14 (*P* < 0.05). This indicated that CCI was an effective model to evaluate NP in vivo and inhibiting or activating the P2Y6 receptor would cause alleviated or aggravated NP analyzed by the PWT and TWL tests.

### 3.2. Manipulation of the P2Y6 Receptor Led to Changes in Marker of Spinal Microglial Activation in CCI Rat Models

**Figure 2:**
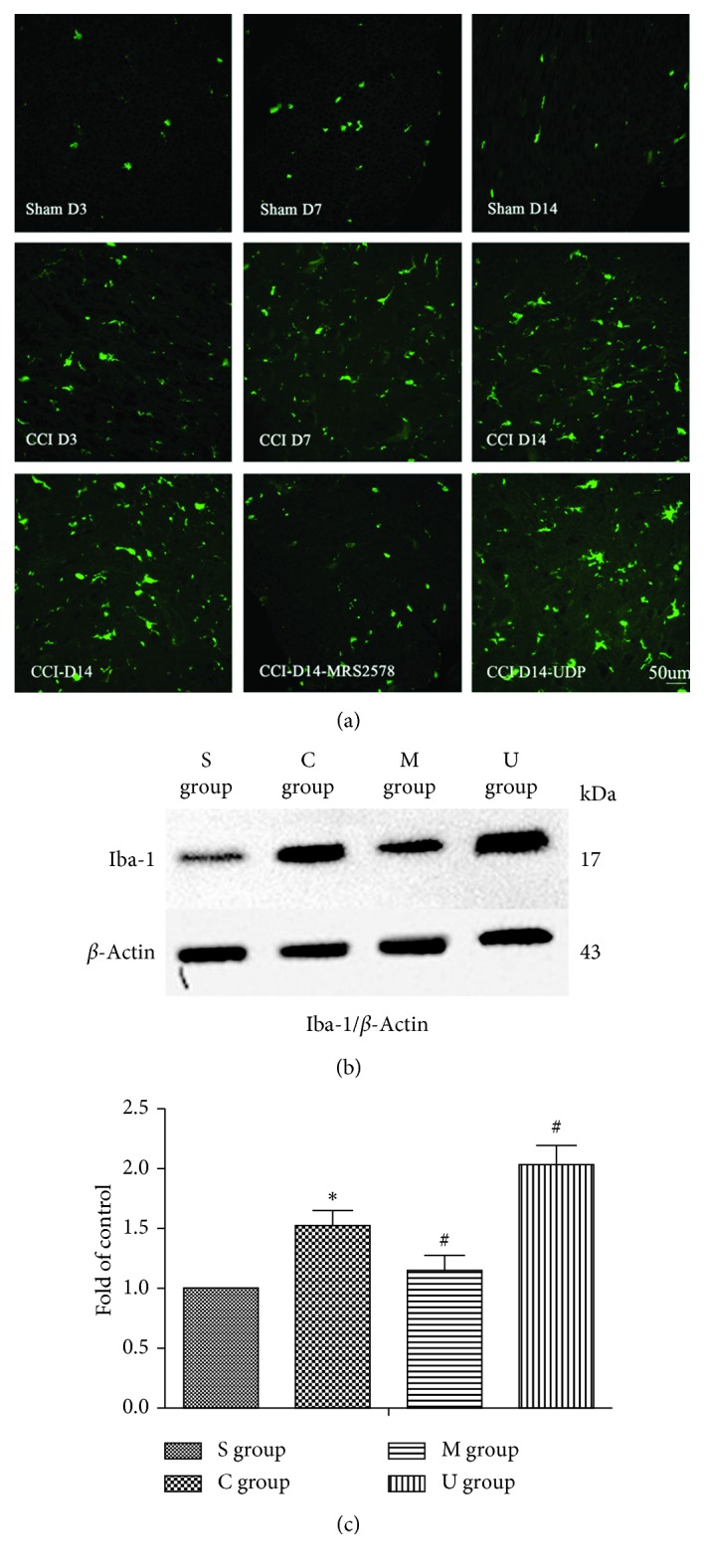
Manipulation of the P2Y6 receptor led to changes in marker of spinal microglial activation in CCI rat models. (a) Representative immunostaining pictures showing the expression of Iba-1 in the L4-5 spinal dorsal horn on days 3, 7, and 14 postsurgery between the CCI and sham group, as well as Iba-1 immunostaining on day 14 among CCI, MRS2578 + CCI, and UDP + CCI. (b) Expression of Iba1 protein in the spinal cord of sham, CCI, MRS2578 + CCI, and UDP + CCI rats on day 14 after surgery quantified by mean ± SEM of integrated optical density (IOD) of Iba1/*β*-actin in rats on day 14 after surgery, *n*=4. S: sham operated rats; C: CCI rats; M: CCI rats treated with MRS2578; U: CCI rats treated with UDP. Significance of Iba1 levels were analyzed with one-way ANOVA followed by the Student–Newman–Keuls post hoc test (^*∗*^*P* < 0.05, vs the sham group; ^#^*P* < 0.05, vs the CCI group).

Given that NP was closely associated with microglial activation after nerve injury, we then performed immunofluorescent staining (Figure 2(a)) and western blot (Figure 2(b)) against Iba-1 to determine microglial activation in L4-5 spinal cords. Compared with the sham group, the Iba-1 fluorescence density in the CCI group significantly increased on postoperative day 7 and day 14. In contrast, application of intraperitoneal injected MRS2578 on top of CCI reduced Iba-1 staining to the level of control (sham), whereas treatment of UDP increased the immunofluorescent signals at D14. There results were next confirmed by western blot, by which the expression of Iba-1 was significantly increased in the CCI group in comparison to sham (^*∗*^*P* < 0.05). Meanwhile, application of MRS2578 further decreased the expression of Iba-1, but administration of UDP significantly increased expression of Iba-1 (^#^*P* < 0.05). These results indicated that CCI could successfully activate microglial cells marked by Iba-1, which was prevented by inhibiting P2Y6 but was promoted by activating P2Y6.

### 3.3. P2Y6 Receptor Mediated the Release of IL-6 upon CCI

**Figure 3:**
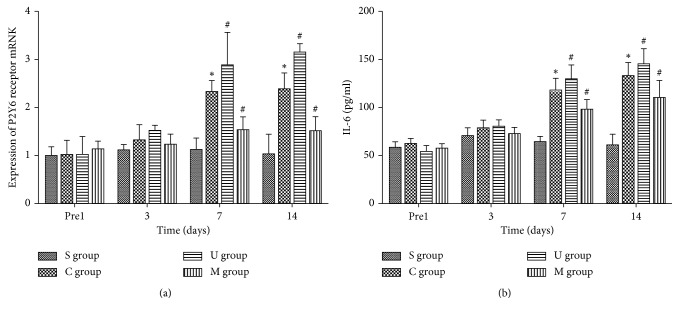
P2Y6 receptor mediated the release of IL-6 upon CCI. (a) Mean ± SEM of IOD of the P2Y6 receptor/*β*-actin in all rats on the mRNA level, *n*=5; (b) mean ± SEM of serum IL-6 concentrations of all rats, *n*=5. S: sham-operated rats; C: CCI rats; M: CCI rats treated with MRS2578; U: CCI rats treated with UDP. Comparison of P2Y6 receptor mRNA and IL-6 among four groups was analyzed with two-way ANOVA followed by Holm–Sidak post hoc analysis (^*∗*^*P* < 0.05, vs the sham group; ^#^*P* < 0.05, vs the CCI group).

After we showed that the P2Y6 receptor participated in NP sensing induced by CCI, we then tested the levels of inflammatory cytokine IL-6, which was implicated in regulating inflammation downstream of P2Y6 [19] and to function in nerve injury-induced NP and spinal neuroinflammation [32, 33]. To assess the relationship of P2Y6 and IL-6 in NP, we measured P2Y6 receptor mRNA expression in L4-5 spinal cord (Figure 3(a)) and the concentration of IL-6 in serum (Figure 3(b)). As shown in Figure 3, the expression of P2Y6 receptor mRNA and release of IL-6 significantly increased in CCI rats on day 7 and continued to day 14 in comparison to sham-operated rats (^*∗*^*P* < 0.05). After peritoneal administration of MRS2578, P2Y6 mRNA and serum IL-6 were obviously inhibited compared to CCI on day 7 and 14 post-operation. On the contrary, peritoneal injection of UDP significantly prompted P2Y6 receptor express and IL-6 release on day 7 and day 14 in CCI rats (^#^*P* < 0.05). These results suggested that P2Y6 mRNA was increased with CCI and antagonist, or agonist of P2Y6 may affect its transcriptional regulation. Also consistent with the above immunofluorescent staining and western blot results, the circulating IL-6 levels were synergistic with levels of microglial activation and pain-sensing behaviors among sham, CCI, CCI + MRS2578, and CCI + UDP groups.

### 3.4. JAK/STAT Signaling Pathway Was Activated in CCI Model Rats

**Figure 4:**
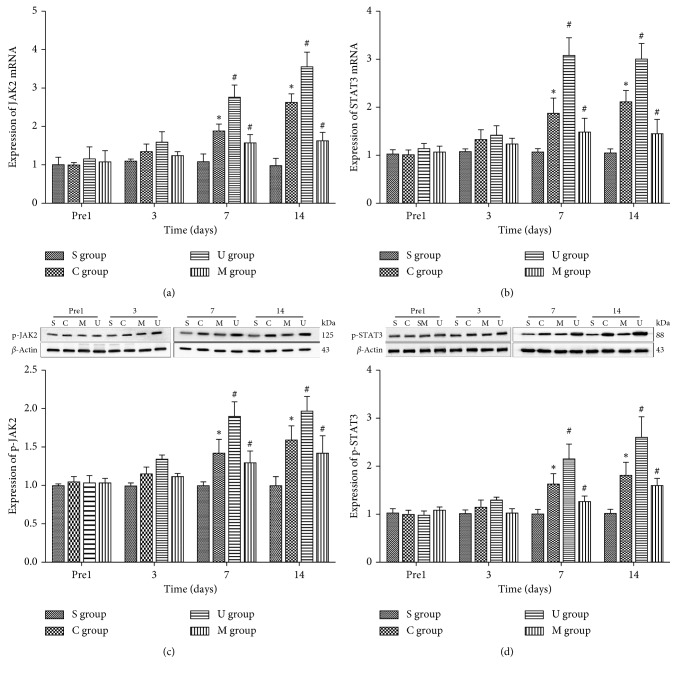
The JAK/STAT signaling pathway was activated in CCI model rats. The mRNA levels of JAK2 (a) and STAT3 (b) were shown with mean ± SEM of IOD of JAK2/*β*-actin and STAT3/*β*-actin in all rats, *n*=5; the phosphorylated JAK2 (p-JAK2) (c) and STAT3 (p-STAT3) (d) levels were shown by the western blot and quantified by mean ± SEM of IOD of p-JAK2/*β*-actin and p-STAT3/*β*-actin in all rats, *n*=5, respectively. S: sham-operated rats; S: CCI rats; M: CCI rats treated with MRS2578; U: CCI rats treated with UDP. Comparisons of JAK2/STAT3 proteins and mRNA among four groups were analyzed with two-way ANOVA followed by Holm–Sidak post hoc analysis (^*∗*^*P* < 0.05, vs the sham group; ^#^*P* < 0.05, vs the CCI group).

In the presence of the fact that increased IL-6 release could trigger the activation of the JAK/STAT pathway via phosphorylation [34], we tested the phosphorylation levels of spinal JAK2 (Figure 4(c)) and STAT3 (Figure 4(d)) by western blot. Furthermore, we also detected the expression of JAK2 (Figure 4(a)) and STAT3 (Figure 4(b)) mRNA by RT-PCR. Our data suggested that CCI significantly increased the expression of JAK2/STAT3 mRNA and p-JAK2/p-STAT3 on day 7 and 14 postoperation (^*∗*^*P* < 0.05). After intervention of MRS2578 in CCI rats, JAK2/STAT3 mRNA and p-JAK2/p-STAT3 levels were significantly downregulated on days 7 and 14 postoperation (^#^*P* < 0.05). However, JAK2/STAT3 mRNA and p-JAK2/p-STAT3 of CCI rats with UDP significantly increased on day 7 and 14 postoperation (^#^*P* < 0.05). These results indicated that the JAK/STAT pathway was activated in CCI-stimulated NP rats by increasing phosphorylation of JAK2 and STAT3, and antagonist of P2Y6 alleviated the phosphorylation levels due to CCI, whereas agonist of P2Y6 further increased the phosphorylation of JAK2 and STAT3. Interestingly, the trend of JAK2 and STAT3 mRNA changes was in accordance with the phosphorylation levels, indicating that the phosphorylation dynamics might be due to substrate availability from transcriptional dynamics.

## 4. Discussion

### 4.1. Role of the P2Y6 Receptor in NP

**Figure 5:**
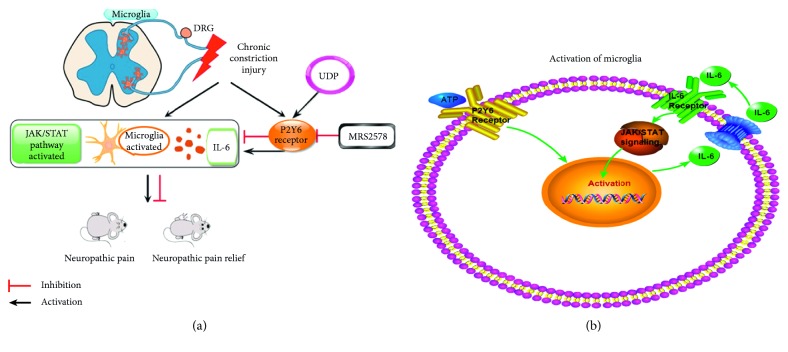
Schematic model of CCI in inducing the activation of microglia and NP. (a) Rats suffered from mechanical allodynia and thermal hyperalgesia after the CCI operation, along with the overexpression of P2Y6 receptors in spinal microglia, which involved in the activation of microglia by prompting the release of IL-6. Accordingly, IL-6 could induce the activation of JAK/STAT signaling in microglia. (b) Activation of P2Y6 resulted in boosted secretion of IL-6, which in turn activated the JAK/STAT pathway, further activating the microglia in secreting more IL-6. It finally formed a positive-feedback loop.

In this study, we have demonstrated that the P2Y6 receptor was involved in the process of microglial activation in NP model rats. Furthermore, we indicated that the stimulation of P2Y6 receptors could trigger sequential reactions for releasing of IL-6 and activating the JAK/STAT signaling pathway in microglia, prompting the neuroinflammation which induced NP (Figure 5). In the CCI model of rats accompanied with tactile allodynia and thermal hyperalgesia, the expression of P2Y6 receptors mRNA was significantly increased. After peritoneal injection of P2Y6 receptor antagonist MRS2578, the hyperalgesia was remarkably relieved. Conversely, the P2Y6 receptor agonist UDP exacerbated the hyperalgesia of CCI rats. Therefore, our results indicated that spinal P2Y6 receptors participated in modulating NP. In accordance with our study, previous study demonstrated that spared nerve injury- (SNI-) induced hyperalgesia was weakened by intrathecal injection of MRS2578 [15]. Similarly, recent study revealed that administration of UDP significantly increased the expression of P2Y6 receptors and Iba-1 in the dorsal spinal cord of naive rats [17]. Moreover, the UDP-treated rats showed persistent pain behaviors without nerve injury, and this kind of UDP-induced hypersensitivity was attenuated by MRS2578 [17]. Taken together, in combination with the administration of the P2Y6 receptor agonist UDP and antagonist MRS2578 on CCI rats, we fully confirmed that the P2Y6 receptor played a crucial role in the development of NP.

### 4.2. Activation of Microglia in NP

Accumulating evidences have well proved the key role of microglia in the pathogenesis of NP [35–37]. The mechanisms of microgliosis and pain following peripheral nerve injury in the animal model of NP may attribute to the release of various proinflammatory cytokines so as to evoke the neuroinflammation [38, 39]. Besides, the activated microglia exhibited marked changes for various genes expression, including innate reactions, external surface receptors, and intracellular signaling factors [40, 41]. We demonstrated the participation of microglia by identifying the spinal Iba-1 up-regulation in CCI rats, and this upregulation was hindered by administration of peritoneal MRS2578 and enhanced by UDP. Our data and existing evidence well suggested that spinal P2Y6 receptors were essential in microglial activation. However, future experiments were needed to understand underlying detailed molecular mechanisms.

The state of microglia is mediated by innate signals via external stimulation and always controlled by a sequence of endogenous transcription factors. It is well accepted that the JAK-STAT signaling pathway plays a vital role in the activation of the microglia [21]. In our experiments, expression of JAK2 and STAT3 was significantly different among four groups of rats, in which tendency of difference in expression was strongly associated with the Iba-1 level in rats. After MRS2578 administration, western blot and PCR data showed dramatic decrease of JAK2 and STAT3 corresponding with Iba-1. Conversely, we witnessed JAK2, STAT3, and Iba-1 increased by using UDP for CCI rats. Previous study also reported that silencing the STAT3 expression could suppress the LPS-induced microglia activation, thus downregulating the expression of harmful factors (iNOS, IL-6, and CCL5) [24]. Another study showed that the expression of the microglial M1-state marker was suppressed after inhibiting the STAT3 activation leading to a relief of local inflammation and hypersensitivity [28]. However, there was evidence showing that JAK/STAT-induced polarization of microglia could bare potential neuroprotective effects [42, 43]. These contradictory reports could be partially explained by the dual functions by this signaling. As mentioned above, both factors of nociceptive (IL-6) and antinociceptive (IL-10) have the ability to trigger the JAK/STAT cascades, yet the transcription of downstream genes differed [24, 43, 44].

### 4.3. Releasing IL-6 Was Associated with the P2Y6 Receptor

In our experiments, our data showed that the IL-6 levels in serum were closely associated with the state of P2Y6 receptors. In the CCI rats, the P2Y6 receptors were overexpressed by nerve injury or hyperactivated by specific agonist UDP, as a result of upregulating the level of IL-6. Furthermore, this kind of upregulation is efficiently prevented by MRS2578. In this condition, we indicated that P2Y6 receptors may control the release of IL-6. Among the P2Y receptor subtypes, existing evidences showed a proinflammatory role of P2Y6 receptors responsible for the IL-6 releasing in the inflammation [19, 45]. Neuroinflammation, inflammation in the nervous system, was stimulated by nerve injury and prompted by the involvement of various cells, factors, and receptors, which lead to the initiation and maintenance of NP [46]. Our present study suggested that the overexpressed P2Y6 receptors, excessive synthesized IL-6, and polarized microglia may all participate in promoting the neuroinflammation by nerve ligation.

## 5. Conclusion

Taken together, IL-6 was the mutual bond to link P2Y6 receptors and the JAK/STAT pathway. We speculate that P2Y6 receptors mediate the activation of JAK/STAT signaling via IL-6 in microglia, during which microglia was polarized and characterized by changing various factors expression. By this way, neuroinflammation could take place keeping the development of NP.

## Figures and Tables

**Table 1:** Primer sequences for RT-PCR amplification.

Gene	Forward primer	Reverse primer
P2Y6R (120 bp)	5′-CGGTCATCGGCTTCTTGCTTCC-3′	5′-CCTTGCTGCGACGCTCTTGG-3′
JAK2 (83 bp)	5′-AAGTGCGTGCGAGCGAAGATC-3′	5′-TGCTGAATGAACCTGCGGAATCTG-3′
STAT3 (176 bp)	5′-CCAGTCGTGGTGATCTCCAACATC-3′	5′-CGCTTGGTGGTGGACGAGAAC-3′
*β*-actin (86 bp)	5′-ATGTTGCAATCCAGGCCGTA-3′	5′-GTGGGTGACACCATCTCCAG-3′

## Data Availability

The data used to support the findings of this study are included within the article.
